# Enzymatic Platforms for Sensitive Neurotransmitter Detection

**DOI:** 10.3390/s20020423

**Published:** 2020-01-11

**Authors:** Sylwia Baluta, Dorota Zając, Adam Szyszka, Karol Malecha, Joanna Cabaj

**Affiliations:** 1 Faculty of Chemistry, Wrocław University of Science and Technology, Wybrzeże Wyspiańskiego 27, 50-370 Wrocław, Poland; dorota.zajac@pwr.edu.pl (D.Z.); joanna.cabaj@pwr.edu.pl (J.C.); 2 Faculty of Microsystem Electronics and Photonics, Wrocław University of Science and Technology, Wybrzeże Wyspiańskiego 27, 50-370 Wrocław, Poland

**Keywords:** biosensor, dopamine, horseradish peroxidase, laccase, polymer, serotonin

## Abstract

A convenient electrochemical sensing pathway was investigated for neurotransmitter detection based on newly synthesized silole derivatives and laccase/horseradish-peroxidase-modified platinum (Pt)/gold (Au) electrodes. The miniature neurotransmitter’s biosensors were designed and constructed via the immobilization of laccase in an electroactive layer of the Pt electrode coated with poly(2,6-bis(3,4-ethylenedioxythiophene)-4-methyl-4-octyl-dithienosilole) and laccase for serotonin (5-HT) detection, and a Au electrode modified with the electroconducting polymer poly(2,6-bis(selenophen-2-yl)-4-methyl-4-octyl-dithienosilole), along with horseradish peroxidase (HRP), for dopamine (DA) monitoring. These sensing arrangements utilized the catalytic oxidation of neurotransmitters to reactive quinone derivatives (the oxidation process was provided in the enzymes’ presence). Under the optimized conditions, the analytical performance demonstrated a convenient degree of sensitivity: 0.0369 and 0.0256 μA mM^−1^ cm^−2^, selectivity in a broad linear range (0.1–200) × 10^−6^ M) with detection limits of ≈48 and ≈73 nM (for the serotonin and dopamine biosensors, respectively). Moreover, the method was successfully applied for neurotransmitter determination in the presence of interfering compounds (ascorbic acid, L-cysteine, and uric acid).

## 1. Introduction

The main role of neurotransmitters, such as dopamine, serotonin, or epinephrine, is to excite, inhibit, and otherwise influence the activity of cells. Any irregularity in the level of neurotransmitters can lead to various serious mental or physical disorders, such as Parkinson’s [1,2], Alzheimer’s [3,4], schizophrenia [5], depression [6], cardiovascular ailments [7], etc. Due to the fact monitoring of these structures is essential in the case of modern medicine, the evolution of biosensors was driven by the need for faster and more versatile analytical methods for application in important areas, including clinical, diagnostics, food analysis, environmental monitoring, industry analysis in complex sample matrices (blood, serum, urine, food), with minimum sample pretreatment. Currently, there is anyfully automated device, such as a biosensor, available on the market that allows for measuring the level of neurotransmitters in real samples.

Serotonin (5-hydroxytryptamine (5-HT)) is directly connected with the regulation of body temperature, mood, sleep, sexuality, and appetite. The human body includes 10 mg of serotonin, with 2% of this being in the central nervous system and any abnormalities in the level of this neurotransmitter may lead to several disorders, including depression, anxiety, and migraines [8,9].

Dopamine (DA) is mainly present in the mammals’ brain and central nervous system. This neurotransmitter is directly responsible for information transmission through the nervous system and any aberration in the level of dopamine may lead to a wide range of disorders and diseases, such as Parkinson’s disease [10].

In the literature, many analytical techniques for catecholamines determination are presented, for instance, spectrophotometry [11,12], chromatography [13,14,15], fluorescence [16,17], flow injection [18], high pressure liquid chromatography (HPLC) [19], and capillary electrophoresis (CE) [20]. However, described procedures are very often time-consuming, require complex pre-treatment steps and the use of toxic substances, and do not permit a continuous analysis. A good alternative for typical analytical methods is an electrochemical biosensor, which provide an inexpensive and easily operable analytical system for the sensitive, rapid, and selective determination of neurotransmitters. Electrochemical approaches are mostly based on using an organic semiconductor for the creation of a suitable matrix for protein anchoring in the bioreceptor part.

Organic semiconductors are interesting candidates for applications at the interface between biological systems and electronics. Macrostructures can be tailored to interact with their aqueous biological surroundings, while at the same time, being able to interface with electronics. The electrically conducting polymers are known to be excellent materials for the immobilization of biomolecules and rapid electron transfer for the fabrication of efficient biosensors. Conducting materials are used to decrease the response time and to enhance the sensitivity of biosensors [21]. Some electrodes modified with semiconducting polymer films for neurotransmitters detection are presented in the literature. Roy et al. modified a glassy carbon electrode (GCE) with an electropolymerized film of *N*,*N*-dimethylaniline (DMA) for DA determination in the presence of ascorbic acid (AA) using square-wave voltammetric (SWV) measurements. It is possible to separate the two peaks corresponding to DA and AA and to detect DA at a very low concentration (μM level) in the presence of AA, whose concentration is close to the physiological level [22]. A sensitive and selective electrochemical method for the determination of DA using a combined electro-polymerized permselective film of polytyramine and polypyrrole-1-propionic acid on a GCE was presented by Zhou et al; the modified electrodes exhibited a detection limit of 100 nM with a linearity ranging from 5 × 10^−6^ to 5 × 10^−5^ M [23]. For simultaneous DA and 5-HT determination, Jiang et al. presented an electrochemical microbiosensor, which was fabricated using electrochemical immobilization of calf-thymus DNA on a carbon fiber electrode (CFE) through an over-oxidized polypyrrole (PPyox) template. A linear response was obtained in the range of 1.0 × 10^−8^ to 1.0 × 10^−6^ M with a detection limit of 7 nM for 5-HT, and in the range of 3.0 × 10^−7^ to 1.0 × 10^−5^ M with a detection limit of 50 nM for DA [24].

In this paper, we present a novel semiconducting polymer-based electrochemical biosensor for dopamine and serotonin detection. The method described here was based on original polymers, which acted as a matrix for enzyme immobilization (stable matrix for a protein anchoring, which also ensured stability during measurements), detection based on an enzyme-dependent redox reaction and electrochemical measurements, and presented a highly sensitive, quick, and simple analytical procedure. However, the main advantage over the other systems known from the literature is the low detection limit in comparison with other systems [25,26,27] and excellent selectivity. The fabricated gold and platinum electrodes modified with semiconducting polymers (poly[2,6-bis(3,4-ethylenedioxythiophene)-4-methyl-4-octyl-dithienosilole], poly[2,6-bis(selenophen-2-yl)-4-methyl-4-octyl-dithienosilole)]), and laccase or horseradish peroxidase (HRP) exhibited exquisite electrochemical behavior and direct electron transfer was achieved. As described in this work, bio-platforms for selective and sensitive neurotransmitter determination directly contribute toward improving quality of life, which is a key priority for global scientific ventures.

## 2. Materials and Methods

### 2.1. Reagents and Materials

Laccase (from *Cerrena unicolor*, EC 1.10.3.2, ≥10 U/mg) and peroxidase (from horseradish, lyophilized powder, 150 U/mg), as well as dopamine hydrochloride (DA), serotonin hydrochloride (5-HT), 2,2′-azino-bis(3-ethylbenzothiazoline-6-sulfonic acid) diammonium salt (ABTS), pyrocatechol, tetrabutylammonium-tetrafluoroborate (TBA-TFB), dichloromethane, uric acid (UA), ascorbic acid (AA), and L-cysteine (CYS) were purchased from Sigma-Aldrich Co (Poznań, Poland). All chemical reagents needed to make buffer solutions were purchased from POCH (Part of Avantor, Performance Materials, Gliwice, Poland). All chemical materials were of analytical grade and were not further purified before use. Synthetic urine CLEANU^®^ was produced by CleanU, Poznań, Poland; CU-25 mL, and it contained creatinine, uric acid, urea, mineral salts, dyes, and water (ingredients reserved due to patent protection).

### 2.2. Apparatus and Procedures

#### 2.2.1. Synthesis and Characterization of Semiconductive Polymers

**Figure 1:**
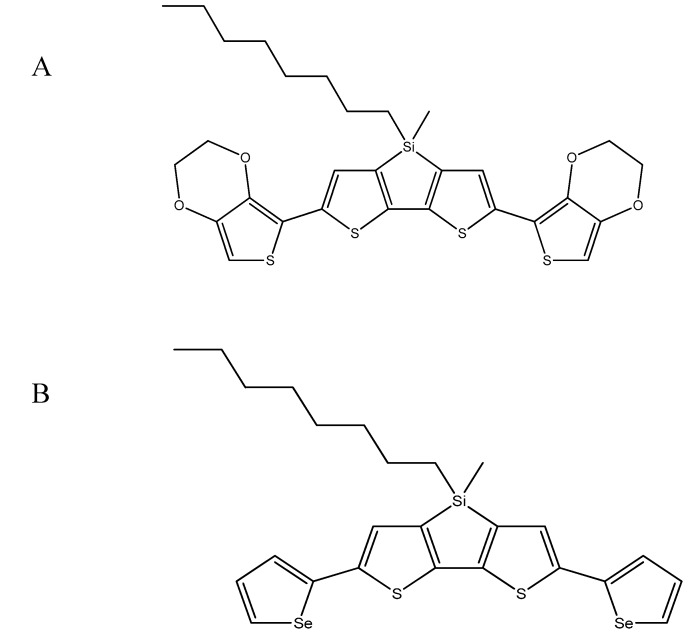
Structure of 2,6-bis(3,4-ethylenedioxythiophen-5-yl)-4-methyl-4-octyl-dithienosilole (**A**) and 2,6-bis(selenophen-2-yl)-4-methyl-4-octyl-dithienosilole (**B**).

**Figure 2:**
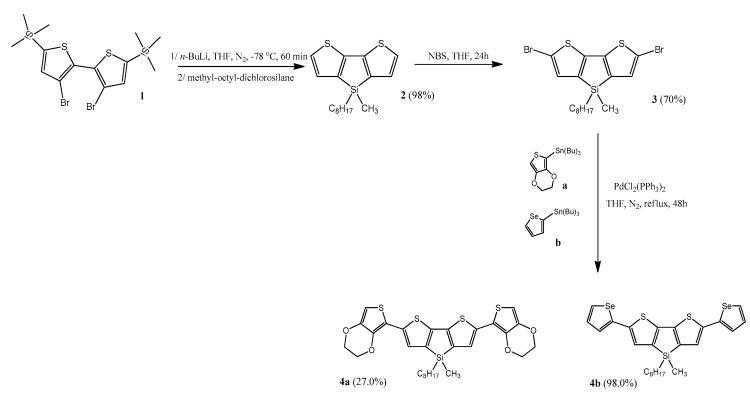
The reaction scheme for the preparation of siloles. NBS: (N-bromosuccinimide), THF: tetrahydrofuran.

Structure of synthesized monomers is shown on Figure 1. The synthesis of 2,6-bis(3,4-ethylenedioxythiophen-5-yl)-4-methyl-4-octyl-dithienosilole (**4a**, see Figure 2) and 2,6-bis(selenophen-2-yl)-4-methyl-4-octyl-dithienosilole (**4b**, see Figure 2) was based on the Stille reaction, i.e., coupling of the organotin compound (**a** or **b**, see Figure 2) with the organic halide (**3**, see Figure 2) catalyzed by the palladium complexes (PdCl_2_(PPh_3_)_2_). The reaction was carried out under nitrogen at 55 °C for 48 h and the medium was anhydrous tetrahydrofuran (THF). The reaction scheme is shown in Figure 2 (entire synthesis process, according to the data [28]).

The increase in the conductivity of polymers can be achieved by the generation of free charge carriers, which are produced using a doping agent. Charge carriers can be generated in several ways: chemically, electrochemically, and via the injection of charge at the interface of the metal semiconductor. Characteristic of the first two techniques is the presence of a doping ion, which accompanies the doping process, stabilizing the cation or anion formed in the π-conjugated system. Other techniques do not require the presence of a doping ion. Although the chemical oxidation process is fairly easy to carry out, it has one major drawback. It is difficult to control, especially in relation to intermediate states of the process, where heterogeneously doped materials are often obtained. The solution to the doping control problem is the oxidation or reduction via electrochemical means. For this purpose, a polarized electrode is used, which is involved in the transfer of electrons, the direction of which is determined by the potential difference between the electrodes. Depending on the electrode polarization, a given compound may undergo oxidation (electron transfer from the molecule chain) or reduction (injection of an electron from the electrode surface onto the chain of the conjugated organic molecule) [29]. The reaction runs in the presence of a basic electrolyte (e.g., TBA-TFB) that provides electrical contact between the electrodes involved in the process. In addition, an appropriate electrolyte, which is introduced into the polymer layer, ensures the polymer’s electro-neutrality.

The electrochemical voltammetric behavior of organic substances, involving as it generally does the generation of charged intermediates, is very sensitive to the nature of the medium in which the experiments are conducted. A variety of homogeneous and heterogeneous processes can be affected by changes in the solvent and/or supporting electrolyte (e.g., TBA-TFB).

According to Fray and Touster [30], TBA-TFB acts like a typical dipolar aprotic solvent to substrates dissolved in it. This can be partly explained due to the high content of salt; however, at the same time, it should also increase the degree of ion-pairing by a mass-action effect. The researchers checked it via an examination of the DC polarogram of anthraquinone dissolved in TBA-TFB. It turned out that TBA-TFB is more sensitive to ion-pairing effects than are hydrocarbons because of the higher electronegativity of oxygen over carbon and the consequent higher charge carried by the two oxygen atoms in the dianion. Due to this, phenol derivatives and other aromatic or heterocyclic compounds appear to be evidently better proton donors in TBA-TFB.

In this study, the electrochemical synthesis of the polymers occurs via electrochemical oxidation (anode process) using cyclic voltammetry. This process takes place in three stages: oxidation of the monomer on the electrode surface with the formation of soluble oligomers in the diffusion layer, polymer settling on the electrode surface combined with the nucleation and chain-growth process, and further polymerization of the layer deposited on the electrode resulting in the formation of longer chains and crosslinked products [31].

#### 2.2.2. Modification of Electrodes

**Figure 3:**
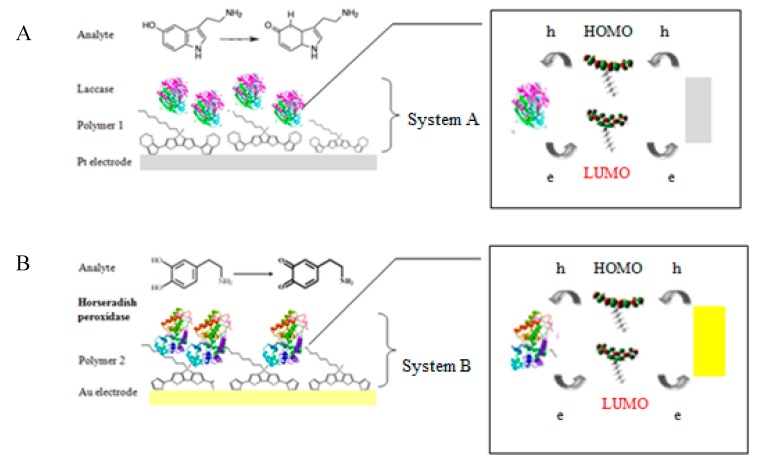
Scheme of (**A**) measuring system A: Pt-E/bisEDOTDTSi/Lac and (**B**) system B: Au-E/bisSeDTSi/Pox. HOMO: highest occupied molecular orbital, LUMO: lowest unoccupied molecular orbital.

Due to the construction of two different biosystems, two different modified electrodes were prepared (Figure 3). The platinum electrode (Pt electrode, diameter 3 mm, produced by BASi (Bioanalytical Systems, Inc., USA (distributor – perfectlab, Warsaw, Poland); model: MF-2013) and the gold electrode (Au electrode, diameter 2.5 mm, produced by BASi, model: MF-2014) were polished before the experiment with 3-µm fine diamond polish and rinsed thoroughly with double-distilled water. The prepared electrodes were modified with a thin layer of polymeric films and enzymes. The platinum electrode for 5-HT determination was modified with a thin layer of poly[2,6-bis(3,4-ethylenedioxythiophene)-4-methyl-4-octyl-dithienosilole] (polymer 1, bisEDOTDTSi) and laccase (system A). The gold electrode was modified with a layer of poly[2,6-bis(selenophen-2-yl)-4-methyl-4-octyl-dithienosilole] (polymer 2, bisSeDTSi) and horseradish peroxidase, which was used for DA detection (system B). All electrochemical measurements, as well as the electropolymerization of monomers, were carried out using a potentiostat/galvanostat AUTOLAB PGSTAT128N (serial nr. AUT84866; Utrecht, The Netherlands) with GPES software (version 4.9) at room temperature under the oxygen saturation conditions. Additionally, for all measurements using the standard three-electrode system—combining a working electrode (Au, Pt), a silver-silver chloride reference electrode (Ag/AgCl), and a coiled platinum wire as the counter electrode—was used. An electrosynthesis of poly[2,6-bis(3,4-ethylenedioxythiophene)-4-methyl-4-octyl-dithienosilole onto the Pt electrode surface and poly[2,6-di(selenophen-2-yl)-4-methyl-4-octyl-dithienosilole] onto the Au electrode were executed using a cyclic voltammetry (CV) technique in a potential range 0.0–1.7 V versus Ag/AgCl for 15 cycles, at a scan rate of 50 mV/s, where both monomers (concentration: 1 mM) were dissolved in an acetonitrile solution containing 0.1 M an electrolyte solution of tetrabutylammonium-tetrafluoroborate (TBA-TFB).

In order to visualize the topography of the surface and to determine the polymer films’ structure, atomic force microscopy (AFM) measurements were performed using a Bruker MultiMode V microscope (Labsoft, Warsaw, Poland). The measurements were performed in tapping mode in air-ambient conditions (25 °C and 35% relative humidity) and at a scanning speed of 3 μm/s. The standard etched rotated silicon tips were used with a tip radius <10 nm and nominal resonance frequency of 300 kHz for the cutting edge.

Furthermore, the deposition of polymeric films was investigated using the scanning electron microscopy (SEM). The polymers were applied directly on the carbon tape and were observed using a JEOL JSM-661OLV (GmbH, Freising, Germany) at 16 kV of beam voltage. The measurement results were averaged and the standard deviation was calculated.

For the engineering of effective biosensors, it is essential to select a suitable immobilization method that will ensure appropriate performances of a biosensor, such as good operational and storage stability, high sensitivity, high selectivity, short response time, and high reproducibility. Knowledge about the biological element or recognition receptors is important due to the biosensor’s construction because the receptor plays a key role in the successful determination of the desired compound. Enzymes possess a high ability for the specific recognition of substrates and to catalyze their specific reaction. However, the species are sensitive to environmental changes, such as temperature or pH value. To minimize this problem, during the biosensor construction, enzymes can be immobilized on the special surface, which should maintain the high enzymatic activity of the protein and stabilize the biomaterial. Such platforms are often an organic material, such as a semi-conductive polymer. Due to this fact, immobilization of both enzymes, i.e., laccase and horseradish peroxidase, onto prepared matrixes based on semiconductive polymers was executed using physical adsorption (2 h) with a crosslinking agent, namely glutaraldehyde. Additional use of the glutaraldehyde for cross-linking permitted covalent bonds creation [32].

Often, the conjugated heterocyclic units are used in the sensor fabrication as electron mediators to improve the contact of the active center of the enzyme with the surface of the electrode. While transferring the electrical charge, polymers serve as a suitable microenvironment for the immobilization of protein and as a transducer [33,34,35]. The interlaced conjugated heterocyclic derivative is expected to facilitate the electron transfer, thereby enhancing the sensor sensitivity. Charge carrier (hole—h^+^ and electron—e^−^) transfer (CT) reactions are necessary in biosensors. The redox-active proteins, such as laccase or HRP, is based on the transfer of charge carriers by hopping and/or long range tunneling [36]. Due to the potential for oxidation or reduction processes that are suitable in a biosensor, both types of CT can be transferred to/from the enzyme. According to the theory, the holes and electrons are transported through the highest occupied (HOMO) and lowest unoccupied molecular orbitals (LUMO). Furthermore, organic compounds (e.g., poly(3,4-ethylenedioxythiophene) with a low ionization potential (below 5.0 eV) are p-type semiconductors [37] and have advanced stability due to the exclusion of oxidative-damage-based inactivation [38]. In our research, poly(2,6-di(3,4-ethylenedioxythiophene)-4-methyl-4-octyl-dithienosilole) was a p-type semiconductor (ionization potencjal (IP) = 4.47 eV, electron affinity (EA) = 3.76 eV) and poly(2,6-di(selenophen-2-yl)-4-methyl-4-octyl-dithienosilole) may be an n-type semiconductor (IP = 5.37 eV, EA = 3.62 eV). We have proposed that holes from the electrode can reversibly reduce dopamine/serotonin. The hole-hopping to dopamine/serotonin is possible when the ionization potential of the organic semiconductor is low, near 5.0 eV. In addition, electrons from the oxidized neurotransmitters may be transferred by the long-range direct tunneling mechanism (when the EA of the n-type semiconductor is around 3.6 eV) [39].

After the procedure, modified Pt-E/bisEDOTDTSi/Lac and Au-E/bisSeDTSi/Pox electrodes were obtained and stored at 4 °C when not used. The described modified electrodes were catalytically active for 60–80 reaction cycles.

#### 2.2.3. Electrochemical Measurements

The electrochemical determination of serotonin and dopamine was achieved using CV (to present the whole redox process, with the applied potential in the range of −0.2 to 0.8 V) and differential pulse voltammetry (DPV) (use for linear range determination, with the applied potential in the range of −0.2 to 0.8 V) techniques. The gold electrode for dopamine detection, unmodified or modified with a thin polymer film of 2,6-di(selenophen-2-yl)-4-methyl-4-octyl-dithienosilole and horseradish peroxidase, was used as a working electrode in a typical three-electrode system. In the second set-up, the platinum electrode for serotonin determination, unmodified or modified with poly[2,6-bis(3,4-ethylenedioxythiophene)-4-methyl-4-octyl-dithienosilole] and laccase, was performed with the CV technique with a potential range of −0.2 to 0.8 V. In the case of serotonin determination, only the CV technique was used for presenting the redox cycle and a linear range because of the weak oxidation signal, which is more visible in CV measurements. All the electrochemical measurements were performed at a scan rate of 50 mV/s for 20 cycles at room temperature with air-opened conditions due to checking the possibility using such systems in electronic devices in the future.

### 2.3. Electrochemical Determination of Dopamine and Serotonin

DA and 5-HT detection was determined using electrochemical techniques in an 8-mL cell. The DPV measurements for DA were provided in the same potential range (−0.2 to 0.8 V) versus Ag/AgCl. After equilibrating the electrochemical systems at the initial potential for 10 s, voltammograms were obtained via scanning the potential. Then, the solution was consecutively replaced by 8 mL of a fresh substrate (DA or 5-HT). A concentration of DA in substrate solution was prepared by dissolving DA in 0.1 M phosphate-bufferd saline (PBS) buffer at pH 7.0 (the same conditions as during the immobilization of horseradish peroxidase) and it was investigated in the range of 0.1–200 μM. The concentration of serotonin was checked in the same concentration range (0.1–200 μM). In both cases, the current response was proportional to the proper concentration.

### 2.4. Influence of Interfering Substances

Interfering substances (ascorbic acid (AA), uric acid (UA), L-cysteine (CYS), and a mix of all interfering substances) at a concentration of 50 μM were added to dopamine and serotonin standard solutions at concentrations of 1, 50, and 100 μM to check the interferences in deficiency, balance, and excess. The mentioned species were mixed each time with epinephrine solutions in a volume ratio of 1:1.

## 3. Results and Discussion

### 3.1. Characterization of Polymers

The key step in a biosensor design is the immobilization of the biological material on a suitable matrix, which should not only effectively bind the biomolecule on its surface, but also prevent the weakening of its catalytic activity and ensure stability during measurements as long as possible. The use of biocatalysts immobilized on solid substrates is much more efficient than the use of the proteins in their native form. The immobilization increases the enzyme’s thermal stability and resistance to chemical denaturing agents, and also prevents protein aggregation [40]. The crucial problem in the design of enzymatic electrodes is to enhance the speed and reversibility of the charge transfer between the enzyme and the electrode.

Conducting polymers have aroused wide interest as materials that act as matrix binding molecules and are used to increase the stability, speed, and sensitivity of various biomedical devices [41].

**Figure 4:**
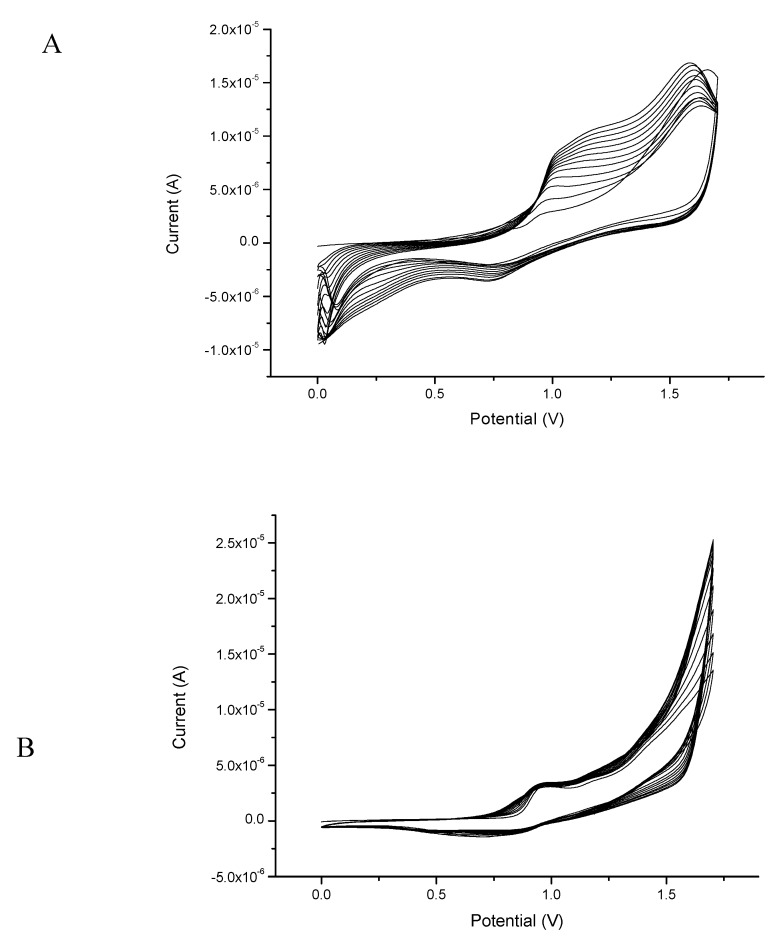
Cyclic voltammograms of investigated monomers: (**A**) electropolymerization of 2,6-bis(3,4-ethylenedioxythiophene)-4-methyl-4-octyl-dithienosilole and (**B**) electropolymerization of 2,6-bis(selenophen-2-yl)-4-methyl-4-octyl-dithienosilole (monomers concentration: 1mM) in 0.1 M tetrabutylammonium-tetrafluoroborate (TBA-TFB). Measurement conditions: scan rate 50 mV/s, Ag/AgCl—reference electrode, 15 cycles.

To synthesize both polymers based on dithienosilole, a CV technique in the potential range of 0.0–1.6 V for 10 cycles was used to obtain optimal polymer layers onto Au and Pt electrodes. Figure 4A,B presents the results of the electropolymerization of both monomers. The voltammograms exhibited the successful electrodeposition onto the electrode surface: 2,6-bis(3,4-ethylenedioxythiophene)-4-methyl-4-octyl-dithienosilole on the Pt electrode and 2,6-bis(selenophen-2-yl)-4-methyl-4-octyl-dithienosilole on the Au electrode.

The used polymers are obtained from synthesized semiconducting monomers [28,42,43]. Their energy gaps, ionization potentials, and electron affinity are in semiconductor-specific ranges. From our previous study, the results clearly show that it is not necessary to use doping in compounds obtained in our laboratory to ensure the transport of electrons between the active center of the protein and the electrode [44,45]. This fact forms the basis of our research. The polymers obtained by us show long-term stability under atmospheric conditions without losing their semiconducting properties. They do not require special storage, which increases the attractiveness of the sensor itself and its competitiveness with other devices.

The electrochemical synthesis of polymers (which are heterocyclic-ring-containing compounds) was carried out in the TBA-TFB electrolyte solution, which acted as a mediator, and due to the architecture of the molecule, there was no need for using an additional doping agent [30].

**Figure 5:**
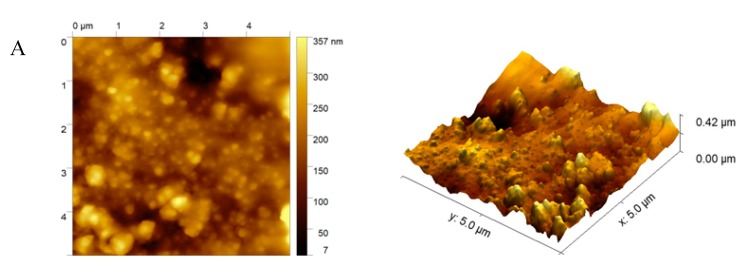

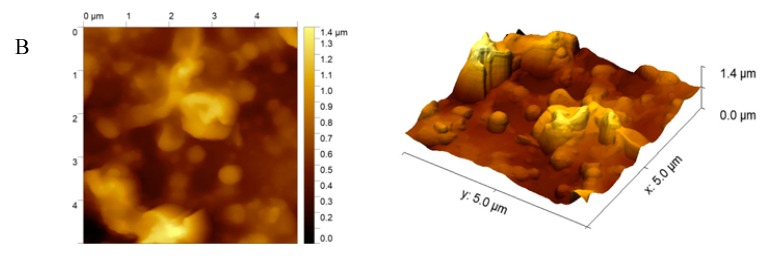
Representative AFM topography maps and surface 3D views of electrode surfaces modified with 2,6-bis(3,4-ethylenedioxythiophene)-4-methyl-4-octyl-dithienosilole (**A**) and 2,6-bis(selenophen-2-yl)-4-methyl-4-octyl-dithienosilole (**B**). Size: 5 × 5 μm.

The morphology of the modified electrodes with the silole derivatives were then investigated using atomic force microscope. The AFM topography maps and surface 3D views of the polymeric films are presented in Figure 5A,B.

As can be observed, the obtained polymer coatings were formed as close-packed layers, which possessed structural integrity. The received morphological images exhibit that the layers based on 2,6-bis(3,4-ethylenedioxythiophene)-4-methyl-4-octyl-dithienosilole (Figure 5A) created more globular compact matrix/layers than the 2,6-bis(selenophen-2-yl)-4-methyl-4-octyl-dithienosilole (Figure 5B). This fact may be connected with the EDOT motif presented in polymer 1 (bisEDOTDTSi), where the disulfide bridges may have been created during the electropolymerization process. Polymer 2 (bisSeDTSi) formed a smoother surface, which could bind an enzyme on its surface less efficiently than in the case of polymer 1.

**Figure 6:**
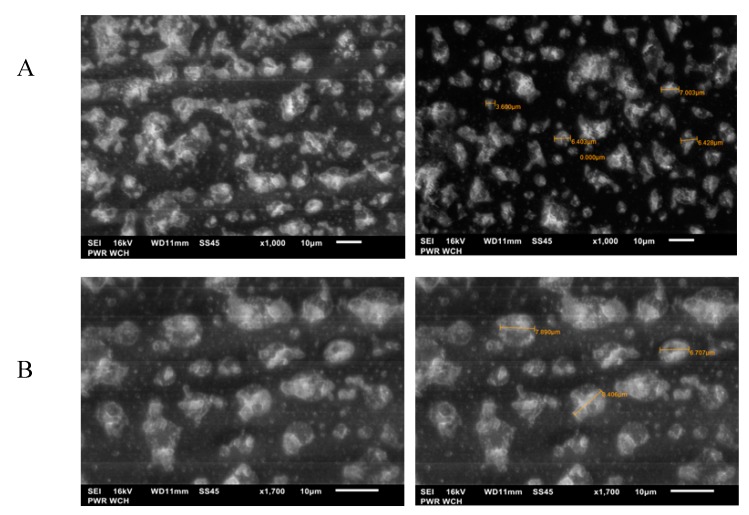
Representative scanning electron microscopy (SEM) images of electrode surfaces modified with 2,6-bis(3,4-ethylenedioxythiophene)-4-methyl-4-octyl-dithienosilole (**A**) and 2,6-bis(selenophen-2-yl)-4-methyl-4-octyl-dithienosilole (**B**). Scale bar: 10 μm.

Moreover, the electropolymerization process and effective deposition of silole derivatives on the surfaces of electrodes was also confirmed using the scanning electron microscopy (SEM) results presented in Figure 6A,B. The SEM images present the polymer film’s creation onto the electrodes without any visible surface defects and with a significantly regular surface. The compounds contained the EDOT moiety in their structure, like in case of bisEDOTDTSi, since various bonds can form during the polymerization process, such as disulfide bridges, van der Waals, and hydrogen bonds; hence, the cylindrical surface shape characteristic for these systems was visible in the SEM images (Figure 6A). The picture of the second polymer (bisSeDTSi), without an EDOT moiety, presented looser packing (Figure 6B) in comparison with bisEDOTDTSi. The diameter of the resulting grains was in the range of 1.5–7 μm, which confirms the creation of different bonds in the polymer structures - in the case of bisEDOTDTSi (Figure 6A) and more comparable diameters of grains in the case of BisSeDTSi (6–8 μm, Figure 6B). These diameters allowed the enzyme molecules to anchor freely into the polymeric films while maintaining their catalytic activity and permit for the rotation to the direct active site of the enzyme toward the substrate. SEM images prove that silole derivatives can be used as a matrix for anchoring the biological elements.

### 3.2. Principle of Electrochemical Measurements and Detection Assays for Neurotransmitters

**Figure 7:**
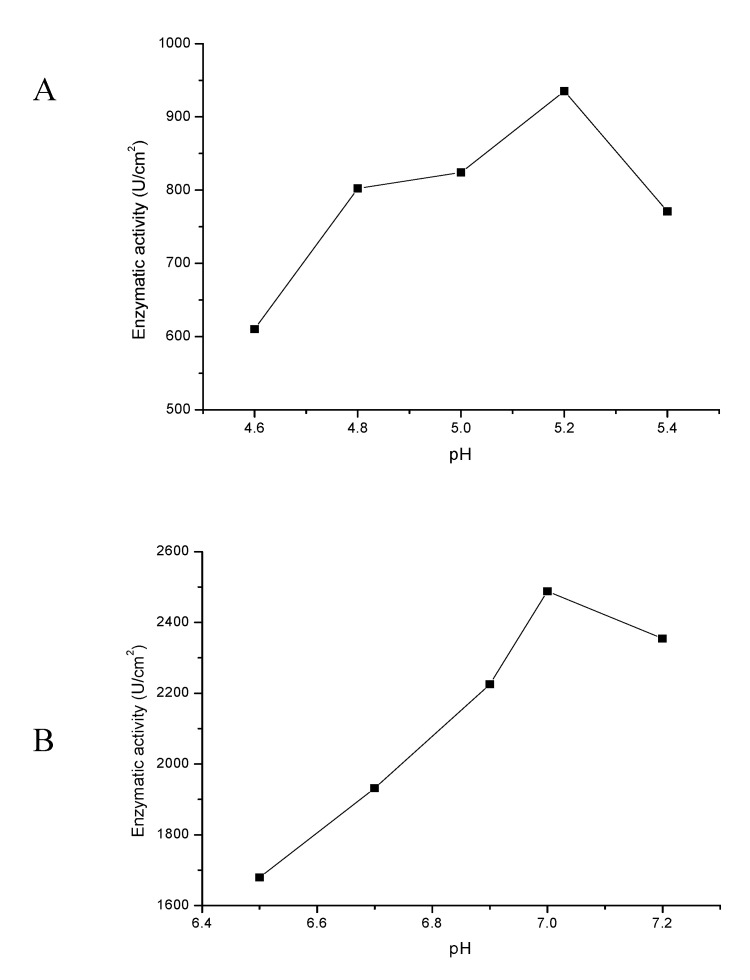
Activity of the immobilized laccase (**A**) and horseradish peroxidase (**B**) at various pH levels.

Frequently, electrochemical procedures are used for neuroscience, e.g., catecholamines identification and determination. The neurotransmitters for measurement have been dissolved in buffers (serotonin in phosphate-citrate and dopamine in PBS), which create a friendly environment in the case of a real sample study, and are the most optimal for enzyme working conditions. As is presented in Figure 7A,B, optimal conditions for laccase and horseradish peroxidase operation are pHs equal to 5.2 and 7.0, respectively, where the enzymatic activity was the highest at room temperature.

Electrodes modified with electrosynthesized polymers, based on described procedure, was stable for several months and stored in 4 °C when not used, which was confirmed with CV measurements.

Both detection systems are based on conducting polymers (CPs), which have become a key platform for electrode modifications. CPs are characterized by the presence of a conjugated π electrons, which give the CPs special properties, such as a low ionization potential, high electron affinities, and a low optical transition energy [46]. CPs, which are easy to synthesize, are cost-effective, and easily modulated by physical changes, have found application as matrixes for binding molecules, which in turn can increase the stability, speed, and sensitivity of various biomedical devices [47,48]. Modification of the electrode improves the performance of the sensor or biosensor, acting as a fast electron transfer mediator between the target molecule and electrode. This results in a negative shift of the peak potential, which in turn allows for better peak separation between different analytes [49]. An accurate and careful design of modified electrodes and electrolyte conditions are important for the achievement of rapid and reversible enzyme–electrode interaction, which depends on the electron transfer reactions. There are two main approaches that allow for the effective coupling between a protein and electrode: first, the direct and unmediated electron transfer, and second, indirect and mediated electron transfer. The direct electron transfer is based on communication between the electrode and the active center of the enzyme without the participation of mediators or other reagents. However, very often, this is difficult to obtain because of the construction of an active center in redox proteins. Frequently, an active center is located deep in a hydrophobic cavity of the molecule, which may result in disturbances of the occurring reaction [50]. In such a situation, when the direct electron transfer is very slow or difficult to achieve, the redox mediators can be used to intensify the rate of electron exchange with the electrode [51]. The application of redox mediators to reach an indirect process possess greater benefits in comparison with a direct transfer method. Additionally, for avoiding the electrode passivation subsequent from the creation of a polymer layer onto the surface of an electrode, using the electron mediators is helpful [52]. In the presented study, we decided to measurements using silole derivatives as a redox mediator in the TBA-TFB environment. Silicone-based structures with long aliphatic chains can anchor the protein, which, due to its good conducting properties, have a valuable impact on the sensitivity of the sensors.

Oxidoreductases, such as laccase and horseradish peroxidase, catalyze the transfer of electrons from one molecule, the reductant (also called the electron donor), to another, the oxidant (also called the electron acceptor). Horseradish peroxidase, due to the low cost and high availability, is one of the most extensively investigated enzymes in the development of biosensors. HRP-based biosensors present the highest sensitivity for a number of phenol derivatives since they can act as an electron donor for enzyme regeneration [53,54]. Nevertheless, due to the direct electron transfer, which causes high current values, the sensitivity of a HRP-based biosensor for phenols decreases. To reduce this problem, HRP should be immobilized on materials in which the direct electron transfer is blocked out to allow for the increase of the sensitivity for phenol compounds (e.g., semi-conductive polymers, like tetraphenylosilane, which has tetrahedral silane in its structure, where the sp^3^ configuration breaks down the conjugated bonds, and as such, the transfer of electrons decreases [55,56]). This enzyme can catalyze the oxidation of a different substrate, e.g., catecholamines [57]. The specific reaction catalyzed by this enzyme converts catechols (e.g., DA) to quinones species (e.g., DA quinone), which are electroactive and can be electrochemically reduced on the electrode surface. The immobilized HRP possesses a high catalytic affinity toward the oxidation of DA, which give the better signals as a result [58].

Laccases catalyzes the oxidation of, for example, ortho- and para-diphenols, aminophenols, polyphenols, polyamines, lignins, and aryl diamines coupled to the reduction of molecular oxygen to water [59,60]. All laccase substrates can be divided in two groups: (I) electron-no-proton donors and (II) electron-proton donors [61,62,63]. Members of the second group (II) include phenols and aromatic amines, for which the formal potentials have a strong dependence on the solution pH. Hydroxylated indoleamines, such as serotonin, can be a substrate for electron transfer reactions that create radical-generating reactions. Serotonin is a potent inducer of redox recycling in the presence of redox-active compounds, such as copper. Serotonin is a good substrate for oxidoreductases and produces reactive oxygen via oxygenases [64]. Chemically and biochemically modified electrodes offer sensitive detection of 5-HT, even in the presence of DA, UA, and AA with very low detection limits.

**Figure 8:**
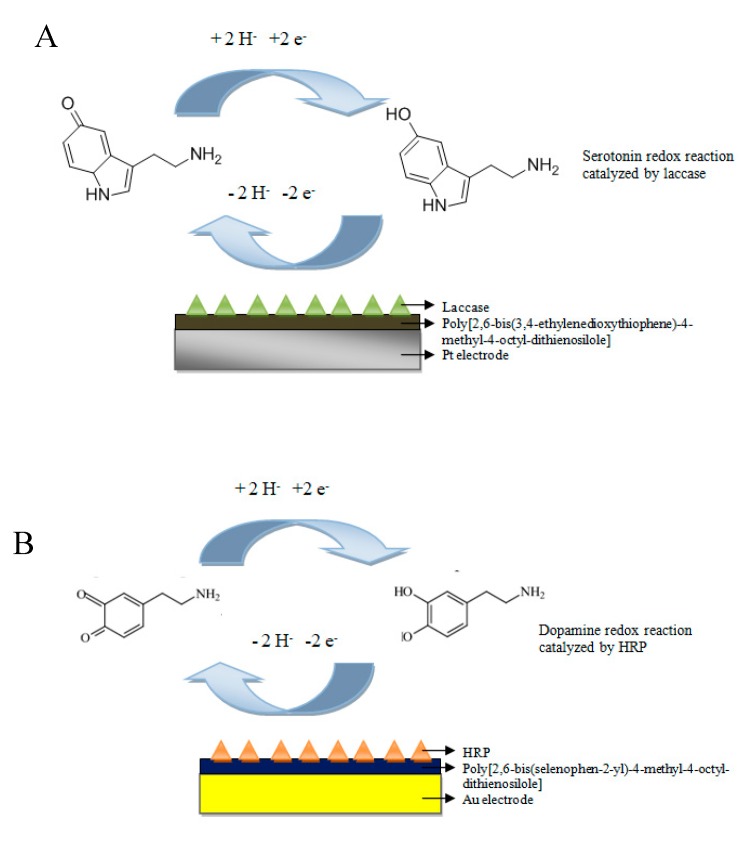
Schematic redox reaction catalyzed by oxidoreductases: (**A**) proposed detection system for serotonin (5-HT) determination and (**B**) detection system for dopamine (DA) determination.

In Figure 8A,B, the redox reactions catalyzed by lacase and horseradish peroxidase are schematically presented. Additionally, the proposed mechanism for the laccase-based reaction of 5-HT oxidation has been suggested.

The first biosystem (A) for serotonin determination was the system using the Pt electrode modified with poly[2,6-bis(3,4-ethylenedioxythiophene)-4-methyl-4-octyl-dithienosilole] (bisEDOTDTSi) and laccase in the presence of a wide range of concentrations of the 5-HT (0.1–200 μM).

**Figure 9:**
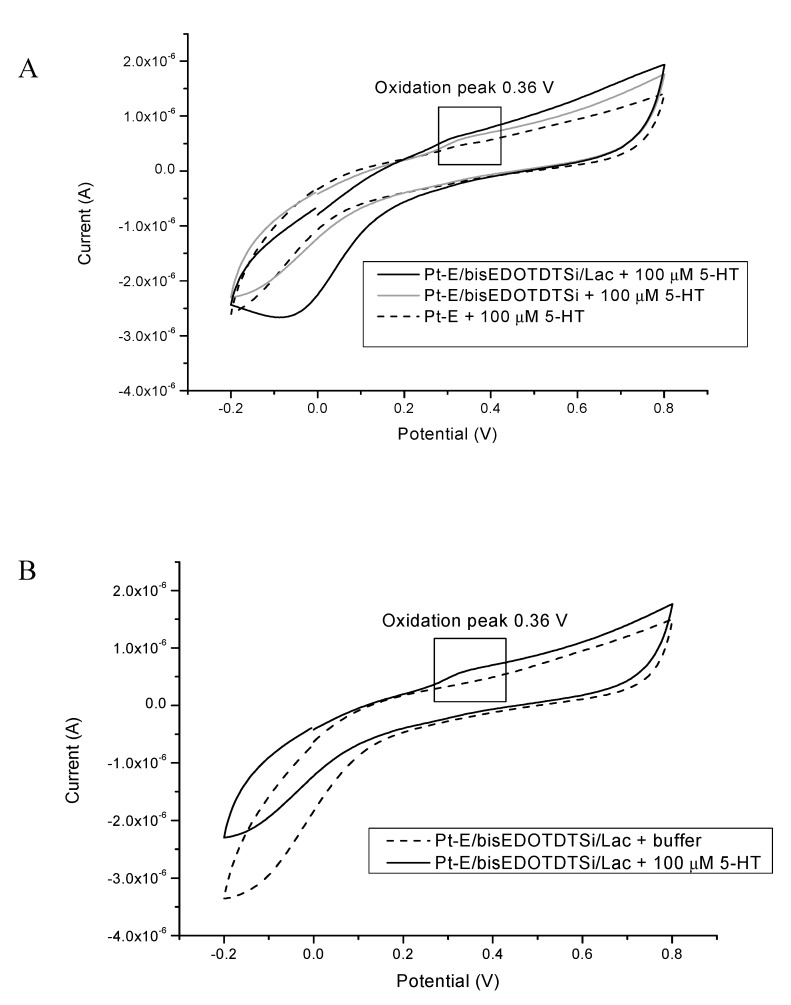
(**A**) Representative cyclic voltammetry (CV) scans of the bare Pt electrode (1), Pt electrode modified with bisEDOTDTSi (2), and Pt-E/bisEDOTDTSi/Lac (3) system in the presence of 5-HT (100 μM) under an applied potential in the range of −0.2 to 0.8 V, scan rate 50 mV/s, versus Ag/AgCl (0.1 M). (**B**) Detection system for Pt-E/bisEDOTDTSi/Lac with and without the investigated sample (5-HT).

Figure 9A shows the CV voltammograms obtained for a bare Pt electrode, Pt electrode modified with bisEDOTDTSi, and the Pt-E/bisEDOTDTSi/Lac complex, recorded in the presence of 100 μM 5-HT. Differential pulse voltammetry (DPV) was a more accurate method for determining the specific analytes in comparison with the CV method, where the low sensitivity makes CV inapplicable for quantitative analysis. However, due to serotonin being quite difficult to measure, it was decided to use the CV technique. The oxidation peak of serotonin at the bare Pt electrode was broad and weak, which associated with very slow electron transfer. An increased peak was visible for the modified polymer electrode; however, as it can be observed, the signal from the oxidation peak was the highest in the presence of laccase, which proves that using the protein in the modified electrode significantly improved the performance of the bioreceptor part. Moreover, the peak potential was shifted to a less positive value (3.7 V), which is characteristic for 5-HT determination (around 3.76 V [65]). Furthermore, an additional measurement for presenting the enzymatic activity of the laccase immobilized on the modified electrode was executed, which was Pt-E/bisEDOTDTSi/Lac only in the presence of a phosphate-citrate buffer (Figure 9B). As shown, buffer did not provide any oxidation peak, with the signal coming directly from the 5-HT.

As it can be observed in the CV results in Figure 9A,B, an electrochemical determination of 5-HT levels is very complicated (weak visibility of 5-HT oxidation peak) because of high sensitivity to other electroactive biomolecules, which can interfere with 5-HT; furthermore, the oxidation products of serotonin can absorb onto the surface of electrode, which disrupts the measurement, and may influence the oxidation peak [49]. Due to this, it was decided to provide only the CV measurements, which gave better signals and showed the whole electrochemical process. Additionally, it allowed for the use of a bare electrode for the determination of serotonin results at a low sensitivity and selectivity due to the above reasons. To resolve these problems, the most effective approach was to choose conductive and selective materials for the modification of the working electrode to improve the measuring sensitivity and selectivity toward 5-HT.

**Figure 10:**
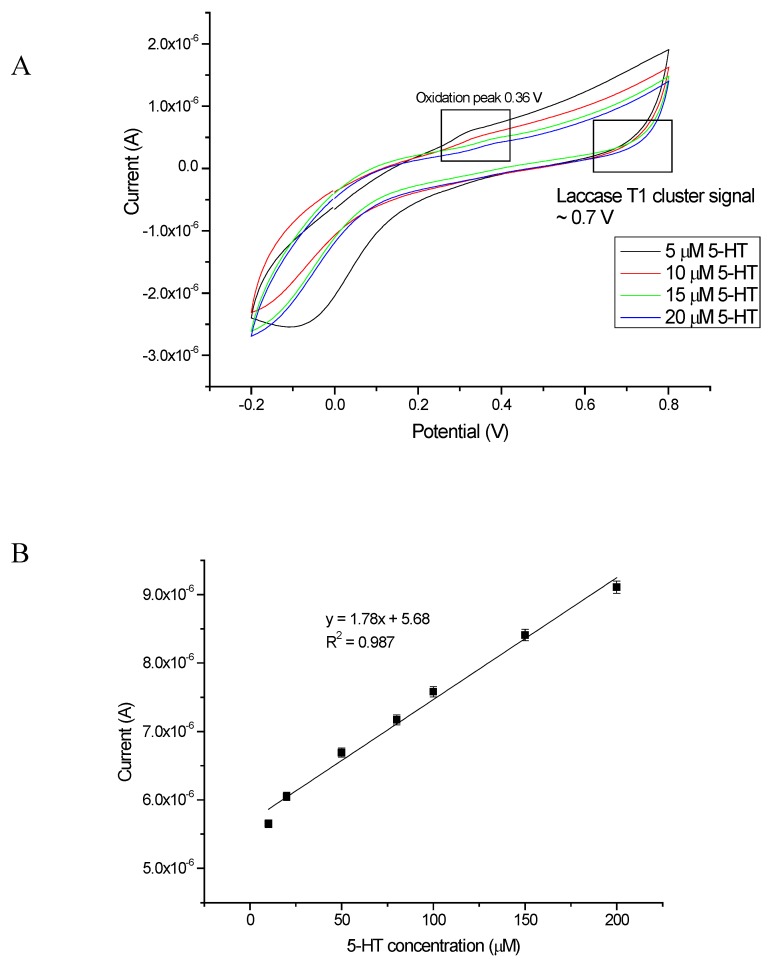
(**A**) Representative CV scans for increasing concentrations of 5-HT (5, 10, 15, and 20 μM) and (**B**) relationship between 5-HT concentration and current (biosensor response).

In Figure 10, the results obtained for bio-system A are presented. As can be observed, the signals responded precisely to the given 5-HT concentration. The signals increased proportionally with the increasing concentration of the examined neurotransmitter. The increased electrical signal was due to the charge (electron) released in the oxidation/reduction process, occurring between the enzyme laccase and the analyte 5-HT. The transfer of electrons between the analytes and the surface of the electrode is a fundamental process occurring in amperometric electrochemistry methods. One of the most promising applications for electron transfer is the use of redox enzymes, which are able to perform selective reactions [66,67].

Direct electron transfer (DET), which occurs between the redox-active enzyme and the electrode surface, causes electronic coupling between the redox protein and electrode. In the third-generation biosensors, DET is associated with the catalytic reaction: substrate $\underset{}{\to }$ product. The redox enzyme acts as an electrocatalyst, simplifying the electron transfer between the electrode and the substrate molecule by involving no mediator in this process [68]. According to this, biosensors based on the DET process present higher selectivity because they are less exposed to interference (they can operate in a potential range close to the redox potential of the enzyme). Although third-generation biosensors exhibit favorable properties, only a few enzymes were found to be capable of interacting directly with an electrode while catalyzing the corresponding enzymatic reaction, e.g., laccase, in a way that could promote DET [69].

Laccase includes four copper atoms: T1, T2, and two T3 due to their spectroscopic properties. With higher potential values, the copper center T1 of laccase can be reduced by phenol-derivative compounds, one-electron redox mediators, and by direct electron transfer from electrodes [61,70,71,72,73]. Additionally, in 2010, it was demonstrated that laccase can be inhibited by self-produced H_2_O_2_ during the electrochemical process [74].

The direct electron transfer to the T1 Cu of laccase takes place at the usual high potential over 0.6 V versus Ag/AgCl [75]. In accordance with the above-described facts, an oxidation mechanism of serotonin catalyzed by laccase was proposed. As it can been observed in Figure 10A, the specific signal of redox of the T1 cluster of laccase was present, as well as an oxidation peak from serotonin (Supplementary Materials; Figure S1A,B, available online).

The biosensor response was linear and proportional as the concentration increases, and at the same time, the current increased. The electrochemical nature of 5-HT was examined in a wide range of concentrations (0.1–200 μM) by employing the CV method (applied potential −0.2 to 0.8 V, scan rate 50 mV/s) in oxygen-saturated conditions for 20 scans (Figure 10B). Figure 10B presents the results of linearity of the biosensor based on Pt-E/bisEDOTDTSi/Lac showing a good linear response to 5-HT in the investigated concentration range with a linear coefficient of R^2^ = 0.987. The slope of the calibration curve was associated with the biosensor’s sensitivity, which was related to the limit of detection (LOD).

One of the most important parameters in construction of a biosensor is the LOD. LOD is the lowest concentration of analyte that can be checked with statistical certainty using the presented technique. In human plasma, the concentration of serotonin is around 14.7 pM [76]. However, 5HT levels and turnover are increased in primary hypertension and certain types of secondary hypertension, such as pregnancy, erythropoietin, and cyclosporine-induced hypertension to even a few dozen nanomol [77].

The LOD was calculated using the following equation:

(1) $$LOD=3.29\cdot \frac{\sigma_{B}}{b},$$

where $\sigma_{B}$ is the standard deviation of the population of blank responses and *b* is the slope of regression line [78]. The limit of detection for the system was equal to 48 nM. The low detection limit demonstrates the ability of the constructed platform to detect low 5-HT concentrations in the case of hypertension-affected patients. This value can be compared with other biosensor systems known from the literature, as seen in Table 1, and is shown to perform much better.

Additionally, the limit of quantification (LOQ) was also determined (calculated using Equation (2)) and it was equal to 73 nM:

(2) $$LOQ=5\cdot \frac{\sigma_{B}}{b},$$

where $\sigma_{B}$ is the standard deviation of the population of blank responses and *b* is the slope of regression line [83]. Furthermore, the sensitivity of the proposed biosensor was found to be 0.0369 μA mM^−1^ cm^−2^. Parameters demonstrating an analytical validation are shown in Table 2.

Another investigated parameter was the standard heterogeneous rate constant (*k_s_*) employed in the Velasco equation (Equation (3)) [84]:

(3) $$k_{s}=1.11D^{1/2}{\left(E_{p}-E_{\frac{p}{2}}\right)}^{-1/2v^{1/2}},$$

where *k_s_* is the standard heterogeneous rate constant, *D* is the diffusion coefficient, *E_p_* is the oxidation peak potential, $E_{\frac{p}{2}}$ is the half-wave oxidation peak potential, and *v* is the scan rate. *k_s_* values were investigated for the Pt-E/bisEDOTDTSi/Lac complex during 5-HT oxidation and for the bare Pt electrode. The results obtained were: for the complex with laccase, *k_s_* = 2.59 × 10^−8^ cm/s, and for the bare electrode, *k_s_* = 6.83 × 10^−9^ cm/s. The difference was attributed to the faster oxidation of 5-HT when applying the enzyme laccase, which catalyzed the oxidation/reduction reaction.

The results presented for the biosystem A were characterized by high sensitivity, a wide linear range, and a low detection limit, which proved that this constructed system may be a very good platform for clinical applications to accurately determine the level of serotonin.

The second biosystem (B) was constructed for dopamine (DA) determination. It was a system using the Au electrode modified with poly[2,6-bis(selenophen-2-yl)-4-methyl-4-octyl-dithienosilole] (bisSeDTSi) and HRP. The measurements were provided using a wide range of DA concentrations from 0.1 µM to 200 µM.

**Figure 11:**
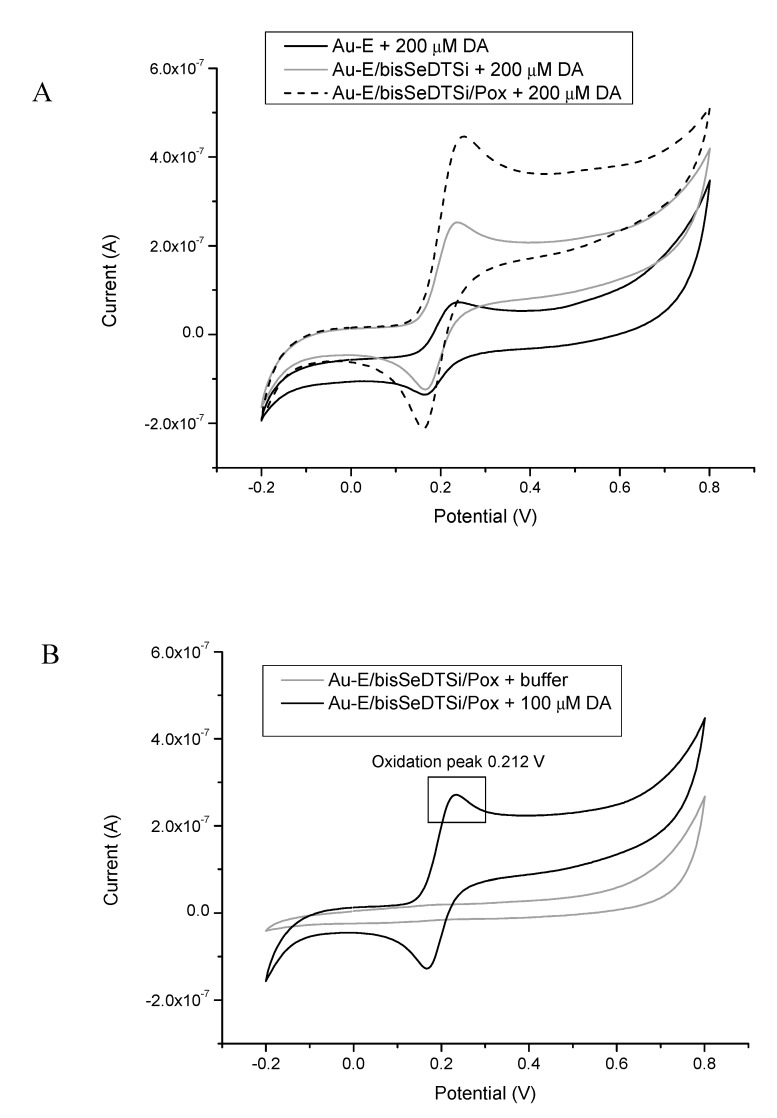
(**A**) Representative CV scans of the bare Au electrode (1), Au electrode modified with bisSeDTSi (2), and Au-E/BisSeDTSi/Pox (3) system in the presence of DA (200 μM) under an applied potential in the range of −0.2 to 0.8 V, scan rate 50 mV/s, versus Ag/AgCl (0.1 M). (**B**) Detection system for Au-E/BisSeDTSi/Pox with and without the investigated sample (DA).

Figure 11 A provides the CV curves of a bare Au electrode, Au electrode modified with bisSeDTSi, and the Au-E/bisSeDTSi/Pox complex, recorded in the presence of dopamine at a concentration of 200 μM. Examination of the DA oxidation peak potential in such various systems should show the significance of using the detection bioplatform, which highly improved the obtained signals. As shown in Figure 11A, the signal from the DA oxidation peak (0.212 V) had the highest response in current value in the presence of the enzyme, which shows that the enzyme catalyzed the reaction and improved the bioreceptor’s parameters. Enzymes, as efficient biocatalysts, represent the most extensively studied receptors used in biosensor devices. These proteins possess a high ability for the specific recognition of substrates and to catalyze their specific reaction. Enzymes are very often used in biosensor construction, and despite their major feature of specificity, they also induce a fast response, which makes them very selective, sensitive, and easy to use. Selectivity is a major requirement for a biosensor technology since these analytical tools should detect only a specific analyte from the mixture. The biological receptor plays a key role in the successful determination of the desired compound. Biomolecular recognition is related to non-covalent specific binding and weak interactions (such as hydrophobic forces, van der Waals forces, or hydrogen bonding) between molecules; in the case of biosensors, one molecule is a biologically active material and the other is an analyte. As seen in Figure 11A, the peaks of oxidation were the highest in the presence of the electrode modified with horseradish peroxidase with a current value of 0.483 µA, which was 6 times higher than signal obtained with using a bare Au electrode (0.08 µA). This proved that the enzyme catalyzed the oxidation reaction efficiently and allowed for obtaining a signal over a wider range of concentrations.

Moreover, to register the enzymatic activity of the horseradish peroxidase immobilized on the modified Au electrode, additional measurement were made using the detection system (Au-E/bisSeDTSi/Pox) without DA in the investigated sample, only in the presence of a PBS buffer (Figure 11B). As demonstrated, the PBS buffer did not influence to the measurements, and the oxidation peak was only found for DA.

**Figure 12:**
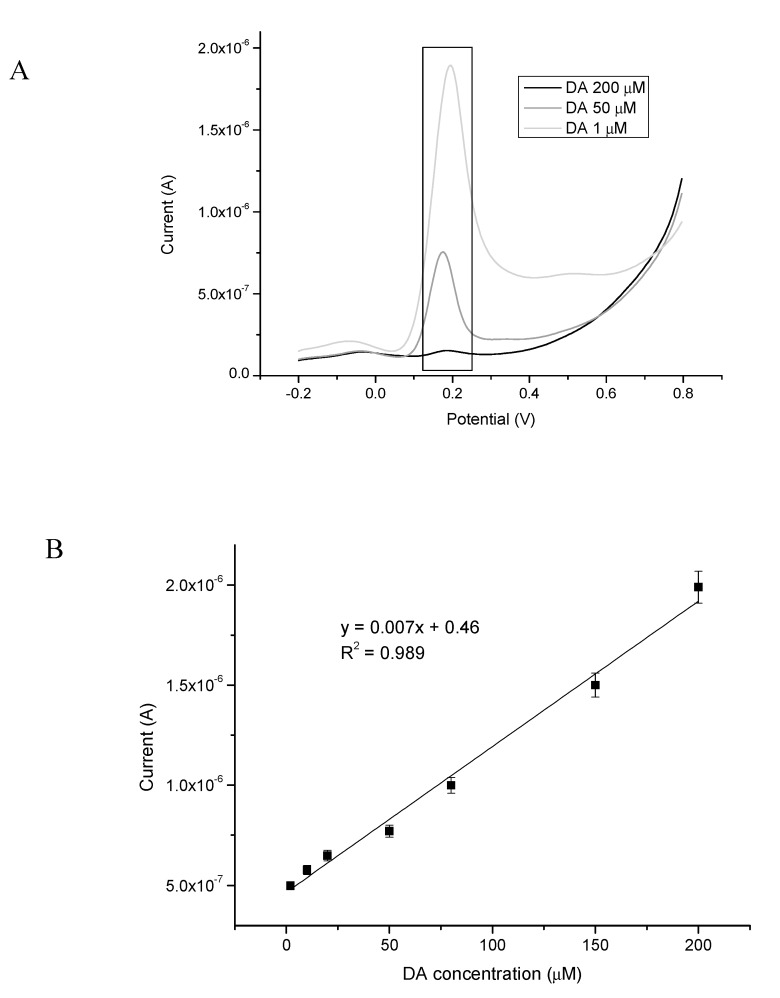
(**A**) Representative differential pulse voltammetry (DPV) scans for increasing DA concentrations (1, 50, and 200 μM), and (**B**) relationship between the DA concentration and current (biosensor response).

The linear range and detection limit of biosystem B was examined using differential pulse voltammetry (applied potential −0.2 to 0.8 V) in oxygen-saturated conditions (Figure 12A). The current signals increased proportionally with the increasing concentration of DA, with each signal corresponding to a given concentration of the examined DA sample. The DPV technique is a more accurate method for detecting analytes in comparison with CV due to the decreased interference of the charging current by taking two measurements, one just before the pulse and the second after a predetermined time [85].

In Figure 12B, the linear relationship in concentration range of 0.1–200 μM of dopamine is presented. For biosensor design, a very important parameter describing the performance of such devices is linearity, which shows the accuracy of the measured response to a straight line [40].

Figure 12B show that the biosensor based on Au-E/bisSeDTSi/Pox had a very good linear response to DA with a linear coefficient of R^2^ = 0.989 for a wide range of concentrations.

The limit of detection for this system (according to Equation (1)) was equal to 73 nM. This value is much lower than the values found for other electrochemical biosensors or sensors for DA determination (Table 3). Moreover, this is a promising value for dopamine biosensor applications. In general, the plasma level of dopamine is equal to the level of adrenaline and fluctuates around 0.054 nM, while in the case of various diseases, such as hypoglycemia, the concentration of dopamine increases sharply [86]. In the face of these considerations, the limit of detection determined in this paper for dopamine corresponds to the concentration of this neurotransmitter in the plasma of a person suffering from dopamine level disorders.

The limit of quantification (based on Equation (2)) was also determined and was equal to 111 nM. Furthermore, the sensitivity of the proposed biosensor was found to be 0.0256 μA mM^−1^ cm^−2^. Parameters demonstrating an analytical validation are shown in Table 4.

According to the Velasco equation (Equation (3)), the *k_s_* value for the Au-E/bisSeDTSi/Pox system during DA oxidation was equal to 6.05 × 10^−6^ cm/s.

### 3.3. Selectivity

**Figure 13:**
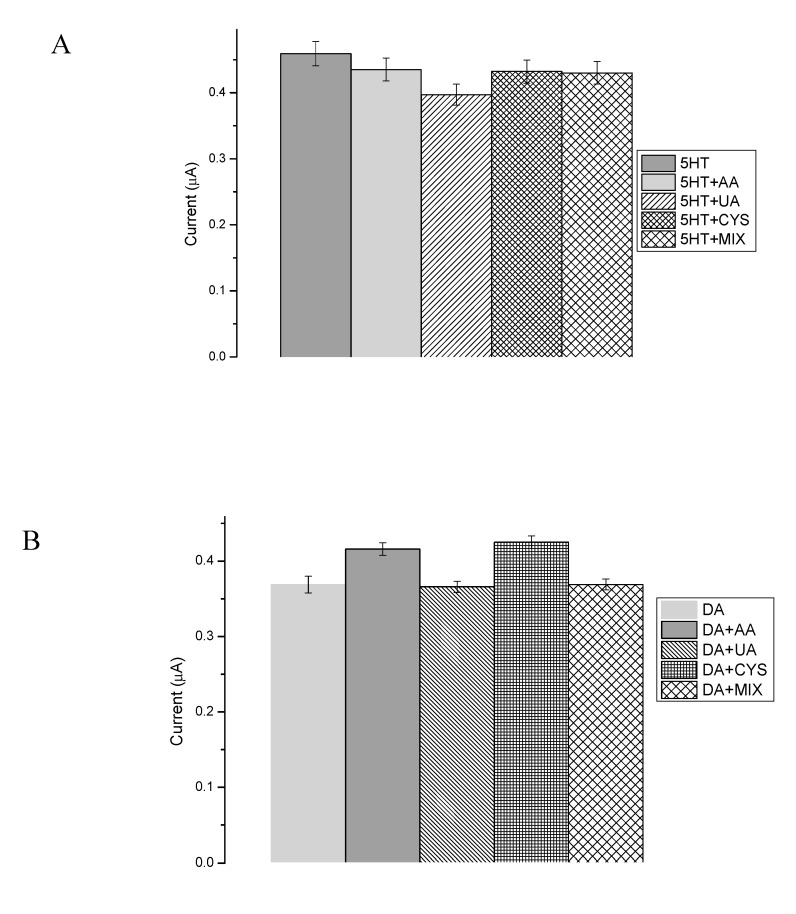
Effect of interfering substances (50 μM) on 5-HT determination (**A**) and DA determination (**B**).

Presented here are the enzyme-based biosensors that were designed for quantitative DA and 5-HT detection in human body fluids’ samples. Body fluids contain a wide range of interfering substances, with the most common being ascorbic acid (AA) and uric acid (UA), which have high concentrations in the human body and possess similar oxidation potentials to 5-HT and DA at bare conventional electrodes [90]. Interfering compounds may have an impact on the oxidation peak potentials and current values during 5-HT and DA examination in human samples, which may lead to an incorrect measurement result. Figure 13A,B presents the influence of interfering substances, such as ascorbic acid (AA), uric acid (UA), cysteine (CYS), and mix of all examined substances during the DA and 5-HT determination. All interfering species (50 μM) were added to the neurotransmitter samples at concentrations of 1, 50, or 100 μM to investigate the impact while carrying out the measurements in the high excess, equilibrium, and deficiency of interfering species. Each tested reagent had a negligible effect (<6.4%) on the peak current of the samples compared to the blank (in case of DA: 0.8% UA, 12% CYS, 11% AA, 2% MIX; and in the case of 5-HT: 12% UA, 3% CYS, 6% AA, 4% MIX). As can be observed in Figure 13A,B, the effect of these species was insignificant, which proved the high selectivity of the designed bioplatforms.

### 3.4. Real Application

The practicability of the introduced study was also estimated with different concentration of DA and 5-HT dissolved in synthetic urine CLEANU^®^, containing, among others, creatinine, uric acid, mineral salts, dyes, urea, and water (exact information reserved according to the patent). The determination of the various concentrations of DA and 5-HT in the synthetic urine based on the CV method (potential range −0.2 to 0.8 V, scan rate 50 mV/s, 20 scans) showed exquisite recovery values (Table 5). The calculations of concentration had been made using the Randless-Sevcik equation (4) [91]:

(4) $$i_{pc}=\left(2.69\times 10^{5}\right)n^{2/3}AD^{1/2}Cv^{1/2}$$

where *i_pc_* is the maximum cathodic peak current, *n* is the number of electrons, *A* is the electrode area (cm^2^), *C* is the redox molecule concentration (mol/cm^3^), and *ʋ* is the potential sweep rate (V/s). *D* is the diffusion coefficient (cm^2^/s). The obtained results proved that the proposed method for neurotransmitters detection was a sensitive, selective, and suitable procedure, which can be used for real sample investigations.

## 4. Conclusions

In summary, a rapid, sensitive, selective, and simple biosensor systems for the detection of dopamine and serotonin was developed using two different systems: system A, based on Pt-E/bisEDOTDTSi/Lac for DA, and system B, based on Au-E/bisSeDTSi/Pox for 5-HT. The electrochemical measurements were carried out by employing the cyclic and differential pulse voltammetry techniques. Both biosensing platforms, based on silole derivatives and oxidoreductases, performed with excellent working parameters (linear range: (0.1–200) × 10^−6^ M in both cases; detection limits: 48 nM and 73 nM, for systems A and B, respectively; and displayed high selectivity and reproducibility). The obtained LOD values for neurotransmitters in this work were lower or similar to others presented in the literature for electrochemical systems. The comparison of both devices shows small differences, with the LOD value being lower in the case of system A (where the CV technique was employed) than in case of bio-system B. However, system B was characterized by a better selectivity and was more stable than system A because DA is easier to detect, in comparison with the complicated 5-HT determination (according to Zając et al. [43] and Section 2.2). However, both detection biosystems proved that all examined interfering substances had a slight effect on the signal during amperometric measurements (≤6.4%), and the obtained biosensors were successfully validated with an adequate recovery using the proposed strategy for biological sample detection. The characteristics of both systems allowed for convenient, stable, simple, and long-term techniques for neurotransmitter detection and they are recommend as excellent analytical biotools. Our preliminary studies presented here may lead to the creation of a sensitive, fast, biocompatible, and selective bio-device suitable for single-use, disposable, in vitro, or in situ applications.

## Figures and Tables

**Table 1:** Comparison of biosensors and sensors for 5-HT detection.

	Biosensor/Sensor	Technique	Linear Range	LOD	Ref.
1	GCE/CNFs	DPV	0.1–10 μM	250 nM	[79]
2	AuNPs/CF-DNA	CV, DPV	0.8–200 μM	800 nM	[80]
3	Fe_3_O_4_NPs-MWCNT-poly(BOG)	DPV	0.5–100 μM	80 nM	[81]
4	RGO/Co_3_O_4_ nanocomposite	CV, DPV	0.1–51 μM	48.7 nM	[82]
5	Pt-E/bisEDOTDTSi/Lac	CV	0.1–200 μM	48 nM	This work

1 Glassy Carbon Electrode (GCE)/Carbon Nanofibers (CNFs); 2 Gold Nanoparticles (AuNPs/Carbon Fiber (CF); 3 Multi-walled Carbon Nanotube (MWCNT)/poly bromocresol green (BOG); 4 Reduced Graphene Oxide (RGO).

**Table 2:** Analytical parameters of the calibration straight of biosystem A. LOD: limit of detection, LOQ: limit of quantification.

Linear Range	LOD	LOQ	R^2^	Slope	SD of Slope	Intercept	SD of Intercept
0.1–200 μM	48 nM	73 nM	0.987	1.78	0.00151	5.68	0.166

**Table 3:** Comparison of biosensors and sensors for DA detection.

	Biosensor/Sensor	Technique	Linear Range	LOD	Ref.
1	PdNP/CNF/C	DPV	1–27.5 μM	200 nM	[87]
2	CNP/f-silicate particles/ITO	DPV	0.05–8 μM	360 nM	[88]
3	RGO/GCE	DPV	0.5–60 μM	500 nM	[89]
6	Au-E/bisSeDTSi/Pox	DPV	0.1–200 μM	73 nM	This work

*2 Carbon nanoparticles (CNPs)/Indium Tin Dioxide (ITO); 3 Reduced Graphene Oxide (RGO)

**Table 4:** Analytical parameters of the calibration straight of biosystem B.

Linear Range	LOD	LOQ	R^2^	Slope	SD of Slope	Intercept	SD of Intercept
0.1–200 μM	73 nM	111 nM	0.989	0.007	0.000238	0.46	0.018

**Table 5:** Results obtained for 5-HT and DA determination based on the proposed methods.

Concentration of Neurotransmitter in Real Sample (μM)	C_detected_ (μM)	C_calculated_ (μM) ^eq. 4^	Recovery (%)	Relative Standard Deviation (%)
Serotonin				
200.00	197.98	186.23	98.99	±3.90
100.00	92.64	105.01	92.64	
50.00	49.87	46.73	99.74	
Dopamine				
200.00	198.89	187.02	99.45	±0.88
100.00	97.69	94.14	97.69	
50.00	49.24	46.09	98.48	

## Supplementary Materials

The following are available online at https://www.mdpi.com/1424-8220/20/2/423/s1, Figure S1A,B: A: A close-up of the laccase T1 cluster signal peak and B: close-up of the 5-HT oxidation signal, due to the Figure 10A.
