# Supplementary material for: Enzymatic Platforms for Sensitive Neurotransmitter Detection
Source: Sensors (Basel). 2020 Jan 11;20(2):423. doi: 10.3390/s20020423 (PMC7014284; doi:10.3390/s20020423)
Supplement: Supplementary file 1 [file sensors-20-00423-s001.pdf]

A

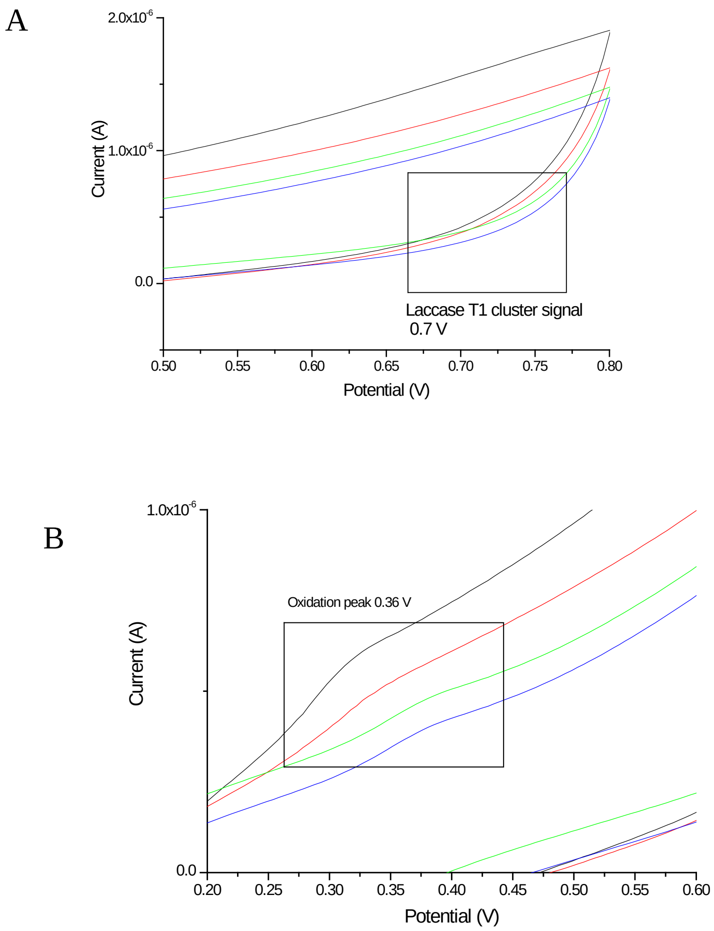

Figure S1. **A,B. A**: A close-up of the laccase T1 cluster signal peak and; **B**: close-up of the 5-HT oxidation signal, due to the figure 10A.
